# Spatiotemporal variability in case fatality ratios for the 2013–2016 Ebola epidemic in West Africa

**DOI:** 10.1016/j.ijid.2020.01.046

**Published:** 2020-04

**Authors:** Alpha Forna, Ilaria Dorigatti, Pierre Nouvellet, Christl A. Donnelly

**Affiliations:** a MRC Centre for Global Infectious Disease Analysis, Department of Infectious Disease Epidemiology, Imperial College London, London, UK; b School of Life Sciences, University of Sussex, Brighton, UK; c Department of Statistics, University of Oxford, Oxford, UK

**Keywords:** Ebola, Case fatality ratio, West Africa, Spatiotemporal analysis, Variogram

## Abstract

**Background:**

For the 2013–2016 Ebola epidemic in West Africa, the largest Ebola virus disease (EVD) epidemic to date, we aim to analyse the patient mix in detail to characterise key sources of spatiotemporal heterogeneity in the case fatality ratios (CFR).

**Methods:**

We applied a non-parametric Boosted Regression Trees (BRT) imputation approach for patients with missing survival outcomes and adjusted for model imperfection. Semivariogram analysis and kriging were used to investigate spatiotemporal heterogeneities.

**Results:**

CFR estimates varied significantly between districts and over time over the course of the epidemic. BRT modelling accounted for most of the spatiotemporal variation and interactions in CFR, but moderate spatial autocorrelation remained for distances up to approximately 90 km. Combining district-level CFR estimates and kriged district-level residuals provided the best linear unbiased predicted map of CFR accounting for the both explained and unexplained spatial variation. Temporal autocorrelation was not observed in the district-level residuals from the BRT estimates.

**Conclusions:**

This study provides new insight into the epidemiology of the 2013–2016 West African Ebola epidemic with a view of informing future public health contingency planning, resource allocation and impact assessment. The analytical framework developed in this analysis, coupled with key domain knowledge, could be deployed in real time to support the response to ongoing and future outbreaks.

## Highlights

• Machine learning (Boosted Regression Trees) was used to predict case fatality ratios for Ebola virus disease.
• The case fatality ratios for Ebola in the West Africa epidemic showed substantial spatiotemporal heterogeneity.
• Analysis using geospatial techniques (kriging and semivariograms) revealed that unexplained variation in the case fatality ratio estimates showed spatial autocorrelation but no temporal autocorrelation.
• In Ebola outbreaks, machine learning and geospatial analysis, coupled with key domain knowledge, could be used to inform the outbreak response.

## Introduction

Ebola virus disease (EVD) is associated with a very high fatality, with 10,884 deaths (among 26,277 cases) reported for the West African epidemic (Qin et al., 2015, Van Kerkhove et al., 2015). Although previously limited to rural communities, recent outbreaks have occurred in urbanised towns and cities in West Africa and more recently in the Democratic Republic of the Congo, further increasing the possibility of fatalities across densely populated communities with limited material and human resources (Barry et al., 2018, Huber et al., 2018, Mbala-Kingebeni et al., 2018, WHO Ebola Response Team, 2015). The case fatality ratio (CFR), defined as the proportion of patients dying from EVD, generally varied between 60% to 90% for known outbreaks (Lefebvre et al., 2014, Van Kerkhove et al., 2015). Robust CFR estimates for entire epidemics and subgroups not only inform the situational awareness during ongoing outbreaks, but are essential for future public health contingency planning, resource allocation and for evaluating the impact of interventions subsequently implemented during outbreaks (Garske et al., 2017).

While published West African CFR estimates are consistent with those reported for other outbreaks, including the most recent outbreaks in the Democratic Republic of Congo (typically 60% to 70% for confirmed and probable cases), CFRs have shown variability over space and time (Barry et al., 2018, Garske et al., 2017). Some analyses have aimed to quantify these variations (Garske et al., 2017, WHO Ebola Response Team, 2015), for example, Garske et al. detected outliers in CFR estimates between districts of residence and treatment centres during the West African epidemic based on individual-level observed survival outcomes (Garske et al., 2017). Spatiotemporal characterisation of other epidemiological parameters, such as transmission rates and mutation rates have also been informative (Carroll et al., 2015, Kramer et al., 2016). For example, Kramer et al. used network models to estimate the spatial process of EVD transmission and found that the probability of transmission between locations depended on distance, population density and the international cross-border importations of EVD cases (Kramer et al., 2016). A strong case has been made that simple spatiotemporal characterisations of such heterogeneities available in real time could have enhanced the national and global response as the West African epidemic unfolded (Koch, 2015).

In Forna et al., a methodological framework built around Boosted Regression Trees (BRT) with adjustments for imperfect model sensitivity and specificity, generated more representative CFR estimates for the West African Ebola epidemic than the crude CFR estimates by imputing survival outcomes for the 44% of the individual cases with missing survival outcomes (Forna et al., 2019). Here, we use a similar BRT imputation framework to investigate the spatiotemporal heterogeneities in CFR for the 2013-2016 West African Ebola epidemic.

## Methods

### Geographical characteristics of the study setting

In this study, the West African region refers to the three most affected countries during the 2013–2016 EVD epidemic, Sierra Leone, Guinea, and Liberia with a combined area covering 429,110 km^2^. The region has been divided into 63 administrative units in total, comprising 14 districts in Sierra Leone, 34 prefectures in Guinea and 15 counties in Liberia (Supplementary Figure 1). For the purposes of this study, districts, prefectures, and counties for Sierra Leone, Guinea and Liberia, respectively, are referred to as districts. Districts were then used as the spatial unit of analysis.

We used GADM (i.e. Database of Global Administrative Areas) data to map the districts of the West African region (GADM (2020)). District-level CFR estimates were geo-referenced, and all 63 districts were included in the analysis. There were 7 districts in Guinea without any EVD cases according to the case database. The Western Urban and Western Rural districts in Sierra Leone were aggregated into a single western district as used in previous analyses (Garske et al., 2017).

### Data sources

EVD cases were reported to the World Health Organisation (WHO) using the viral haemorrhagic fever (VHF) case reporting forms as published in the supplementary information of Garske et al. (2017). In the WHO case reporting system, cases were classified as confirmed, probable and suspected. The epidemiological details of the case definition system have been described in detail elsewhere (World Health Organization, 2014). Here, we report CFR estimates based on all reported cases (confirmed, probable, and suspected). Case classification (confirmed, probable, and suspected) was an important predictor (explained 10.1% of observed CFR variation) of EVD CFR for the West African epidemic and was accounted for in the BRT models used in this study (Forna et al., 2019).

## Spatiotemporal analysis

We used BRT models (Elith et al., 2008) to impute missing survival outcomes and correct for sensitivity and specificity in imputed outcomes in the calibrated BRT model, thus estimating more representative CFRs (i.e. estimates that account for the missingness in survival outcomes unlike the crude estimates that do not account for missingness in survival outcomes) at national and district-level. Briefly, BRT models were calibrated to predict the survival outcomes using the 24 most important predictors of CFR (Forna et al., 2019). Hyperparameters (tree complexity (tc = 27), learning rate (lr = 0.001), bag faction (bf = 0.75) and data partitioning = 0.65) were optimised to maximise out of sample accuracy, allowing us generate district-level and quarterly CFR estimates, along with the relative uncertainty, using a non-parametric bootstrap approach (Forna et al., 2019). Residuals from the adjusted BRT model were also calculated. These residuals were defined as the difference between the observed CFR (i.e. *ib* for spatial and *qb* for temporal observed CFR estimates as defined in section 1.3 and 1.4 in the supplementary material) and predicted CFR estimates adjusted for imputation. Details of the algorithms used for CFR prediction and residual estimation are included in the supplementary material.

Choropleth maps of district-specific median CFRs and their standard deviations were produced for the West African region. Spatial correlation in CFR residuals in the whole West African region was tested for using spatial autocorrelation global Moran’s I index (Lai et al., 2008). We used local Moran’s I index as a local indicator of spatial association (LISA) statistic. The LISA statistic for each district gives an indication of the extent of spatial clustering of similar CFR values around that district and the sum of LISA statistics for all districts is proportional to the global Moran’s I index (Anselin, 1995, Bivand and Wong, 2018). For the LISA statistic, the spatial relationship of the districts was specified using a spatial weight matrix in which neighbours were defined using Delaunay triangulation (i.e. natural neighbours) whereby the centroids of each district define a set of finite points on a plane. For each district centroid, there is a corresponding region (i.e. Voronoi cell) consisting of all points on the plane closer to that district’s centroid than to all of the other centroids on the plane (Deng et al., 2011).

Semivariograms (Srividya et al., 2002) were used to graphically represent the autocorrelation in the estimated CFR at the district-level and in time, at a quarter-level both for the West African region as a whole and for each individual country (Sierra Leone, Guinea and Liberia) (Brooker et al., 2004). Mathematically, the spatial autocorrelation was measured as the semivariance $\left[\gamma \left(h\right))\right]$, which is half the mean squared difference between pairs of district-level CFR values $\;y_{i}$ and $y_{j}$, separated by lag distance (in km) h:

$$\gamma \left(h\right)=\frac{1}{2N(h)\;}\overset{N(h)}{\underset{i}{\sum }}{\left(y_{i}-y_{j}\right)}^{2}$$

where $N\left(h\right)$ is the number of district-level CFR pairs at each lag distance h (Nunes, 2007). In this analysis, the largest lag h was defined as half the maximum distance among all pairs of district-level CFR estimates. A functional form was then fitted to the semivariogram defining three parameters; (i) the sill, which reflects the spatial component of the semivariance (i.e. the level of spatial autocorrelation between district-level CFR pairs), (ii) the range, which is distance at which district pairs are considered to be spatially independent and (iii) the nugget, which represents the small-scale spatial variability or measurement errors during data collection (Lau et al., 2014). Typically, the semivariance between neighbouring districts is expected to be low if there is spatial autocorrelation and gradually increase with the distance between pairs of districts, levelling off at the sill (on the *y*-axis) and range (on the *x*-axis) (Yue et al., 2018). To provide a continuous description of the covariance structure between district pairs, spatial models were fitted to each empirical semivariogram (Jian et al., 1996). Five spatial models were used to examine the spatial relationships including linear, Cauchy, exponential, Gaussian and Matern functions (Cressie, 1993). We used maximum likelihood to identify the spatial model that yielded the smallest Akaike’s Information Criterion (AIC), fitted the overall empirical variogram to each spatial model and used this spatial model for all subsequent analysis (see supplementary material). Where significant spatial correlation was found, we produced continuous maps of the predicted CFR added to the smoothed residuals. The 95% confidence intervals for the sill, range and nugget were obtained using the ‘gboot_variogram’ and the ‘gboot_CI’ functions in the ‘geotoolsR’ R package, refitting the semivariogram to 1,000 non-parametric bootstrap samples of the errors of each fitted semivariogram (Rossoni and Felix, 2017).

Ordinary kriging is a spatial interpolation procedure that assumes that the outcome, in this case the CFR, can be modelled as a continuous spatial process (Carrat and Valleron, 1992). Using the semivariogram model, kriging allowed us to estimate residuals and predicted CFR values at unrecorded districts from the neighbouring districts with recorded estimates using the global neighbourhood approach. Kriging generated a near continuous grid of georeferenced points (26,208 geospatial points on 0.05 × 0.05 km grid) within the West African region. All 26,208 geospatial points are considered in estimating residuals and the predicted CFR for each geospatial point covering districts with both recorded and unrecorded estimates (Davis and Grivet, 1984). The kriged residuals were then added to the kriged predicted CFR adjusted for imputation from the BRT model to produce a best linear unbiased predicted isopleth (i.e. continuous) map of CFR.

As a sensitivity analysis, we investigated the effect of direction on spatial autocorrelation in the CFR estimates. Directional semivariograms were estimated and these were compared to the estimated omnidirectional semivariogram (see supplementary material).

Furthermore, to investigate whether the district effects explained spatial variations, we refitted the BRT model excluding district from the predictors and re-estimated the global and local autocorrelation coefficients (i.e. Moran’s I indexes and their corresponding p-values) for the resulting residuals.

To investigate temporal autocorrelation, we aggregated the CFR estimates and residuals by temporal quarter, estimated temporal semivariograms and fitted models treating differences in time as differences in space. In the temporal semivariogram model, we substituted the lag distance *h* with quarterly time lag *t* and $N\left(t\right)$ is the number of temporal-level (i.e. quarter) CFR pairs at each lag time *t.* Thus, temporal autocorrelation was estimated as $\gamma \left(t\right)$. We used the Durbin–Watson (D-W) test to further quantify and test for temporal autocorrelation (Durbin and Watson, 1971).

## Results

**Figure 1:**
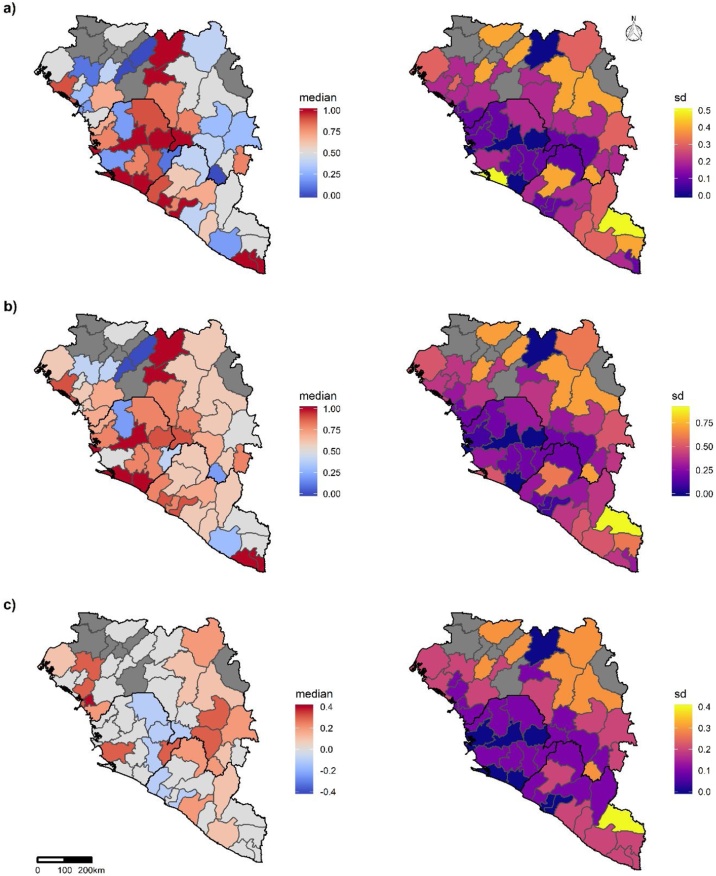
Choropleth spatial distribution of case fatality ratio (CFR) for 2013-2016 Ebola epidemic in West Africa. a) Median predicted CFR adjusted for imputation (and standard deviation) for each district. b) Median observed district-level CFR. Dark grey shading denotes districts for which data were unavailable. c) Median district-level residuals (i.e. observed CFR minus predicted CFR adjusted for imputation). In each case the median is on the left and the standard deviation (sd) is on the right.

Georeferenced district-level predicted CFRs adjusted for imputation and their corresponding standard deviations showed substantial heterogeneity across the districts of Guinea, Sierra Leone, and Liberia (Figure 1a) with district-level residuals showing moderate regional spatial autocorrelation (Global Moran’s I Index = 0.146, p = 0.033). Kailahun (Local Moran’s I Index = −0.685, p = 0.019), Kenema (Local Moran’s I Index = 0.772, p = 0.003) [in Sierra Leone], Beyla (Local Moran’s I Index = −0.908, p < 0.001), and Nzerekore (Local Moran’s I Index=,0.885, p = 0.002) [in Guinea], and Bomi (Local Moran’s I Index = 0.729, p = 0.01) and Grand Cape Mount (Local Moran’s I Index = 0.852, p = 0.003) [in Liberia] were the districts that showed significant local spatial autocorrelation (Supplementary Table 1).

Excluding district from the predictors in the BRT model produced district-level residuals with substantially greater and highly significant global spatial autocorrelation (Global Maron’s I index = 0.348, p < 0.001) and significant local spatial autocorrelation in 10 districts (Supplementary Table 2). One district, Nzerekore, showed significant correlation with/without district as a predictor in the BRT model (Supplementary Table 1).

In Guinea, the predicted CFR adjusted for imputation was highest in Dinguiraye and Dabola and lowest in Tougue (Figure 1a). In Sierra Leone, the predicted CFR adjusted for imputation was highest in a few districts from Freetown in the West Urban area to Kono to the east of the country, and the predicted CFR adjusted for imputation was lowest in Kailahun. In Liberia, the predicted CFR adjusted for imputation was highest in Grand Kru and Maryland and lowest in Sinoe (Figure 1a). Figure 1b shows the observed CFR (observed CFR *ib*, as described in section 1.2, page 4, step 13 in the supplementary material) and Figure 1c shows the corresponding district-level CFR residuals.

**Figure 2:**
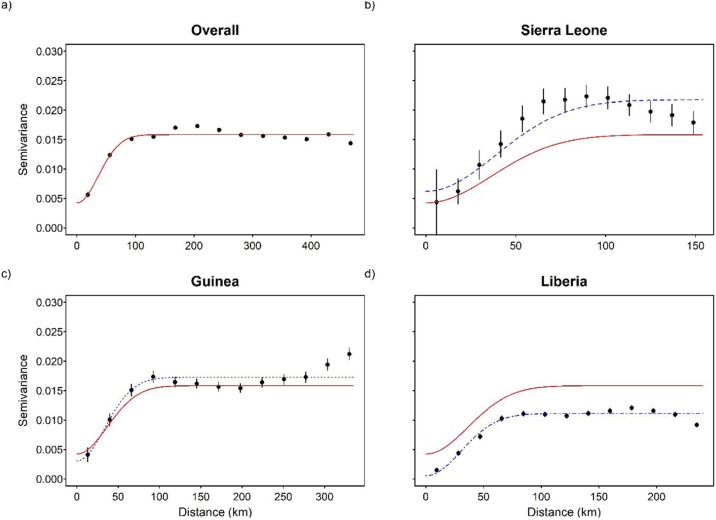
Semivariograms fitted with a Gaussian model to the residuals of district-level CFR adjusted for imputation based on the Boosted Regression Tree model (BRT). The red line is the fitted model for the region as a whole and the blue lines are fitted models for individual countries (Sierra Leone, Guinea and Liberia). Note that the x-axes vary.

Compared to other spatial models (i.e. linear, Cauchy, exponential and Matern), the Gaussian model yielding the smallest Akaike’s Information Criterion (AIC) (Supplementary Table 2) and the most precisely estimated range parameter was subsequently fitted to the empirical semivariograms. Semivariogram analysis showed significant spatial autocorrelation overall (all 3 countries combined) and for Sierra Leone, Guinea and Liberia individually (Figure 2, Table 1). The range parameters were 89.6 km (95% CI, 33.3–99.7 km), 91.1 km (95% CI, 40.9–95.0 km), 82.9 km (95% CI, 30.4–131.8 km) and 78.4 km (95% CI, 34.3–89.6 km) for the region as a whole, Sierra Leone, Guinea and Liberia, respectively (Table 1).

**Table 1:** Parameter estimates for the best-fit Gaussian semivariogram model fitted to the spatial residuals (observed CFR minus predicted CFR adjusted for imputation) from the BRT model.

Spatial parameters	Overall with bootstrap median, (95% CI)	Sierra Leone with bootstrap median, (95% CI)	Guinea with bootstrap median, (95% CI)	Liberia with bootstrap median, (95% CI)
Range (km)	89.6 (33.3–99.7)	91.1 (40.9–95.0)	82.9 (30.4–131.8)	78.4 (34.3–89.6)
Sill (semivariance)	0.0158 (0.0151–0.0166)	0.0218 (0.0206–0.0233)	0.0173 (0.0161–0.0187)	0.0111 (0.0104–0.0121)
Nugget (semivariance)	0.0042 (0–0.0105)	0.0062 (0.0004–0.0120)	0.0031 (0–0.0122)	0.0005 (0–0.0068)

**Figure 3:**
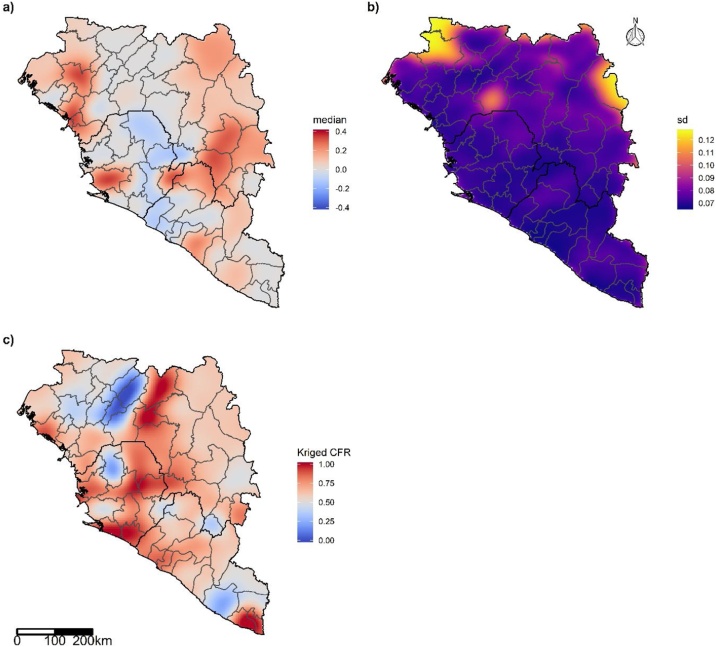
Isopleth case fatality ratio (CFR) map for West Africa of a) median and b) standard deviation (sd) of kriged residuals (i.e. observed CFR minus predicted CFR adjusted for imputation). c) Isopleth map for kriged CFR (i.e. predicted CFR adjusted for imputation plus the kriged residuals).

Because spatial structure was identified across the region (Figure 3a & b), an isopleth map combining the predicted CFR adjusted for imputation and the kriged residuals was used to account for unexplained spatial structure in the data (Figure 3c). On this kriged map, CFR was highest in coastal West African region, from Conakry in Guinea to the Western district (including the capital Freetown) in Sierra Leone and towards Montserrado county (including the capital Monrovia) in Liberia (Figure 3c).

**Figure 4:**
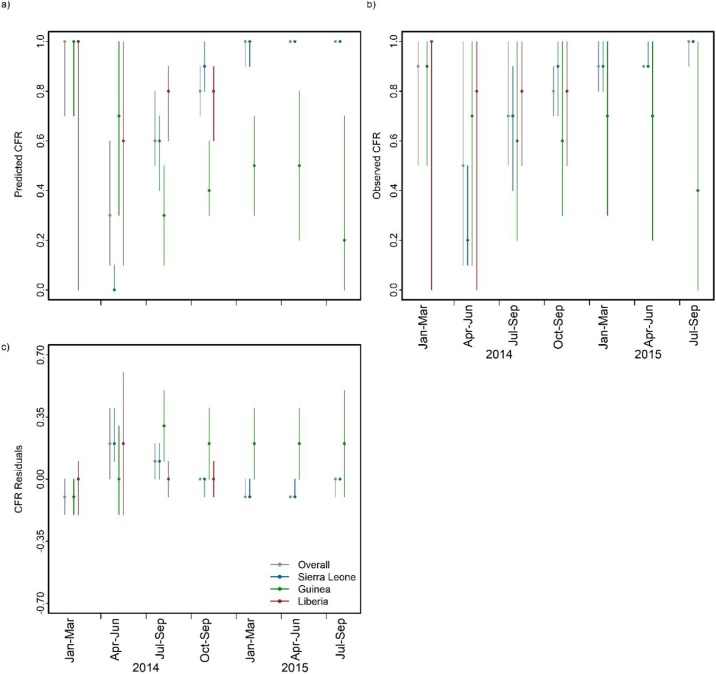
Temporal distribution of case fatality ratio (CFR) for 2013-2016 Ebola epidemic in West Africa. a) Median predicted CFR adjusted for imputation (and 95% CI) for each quarter. b) Median observed quarterly CFR. c) Quarterly residuals (i.e. observed CFR minus predicted CFR adjusted for imputation). Note that there were too few cases in late 2013 and early 2016 to be meaningfully aggregated into quarter.

**Figure 5:**
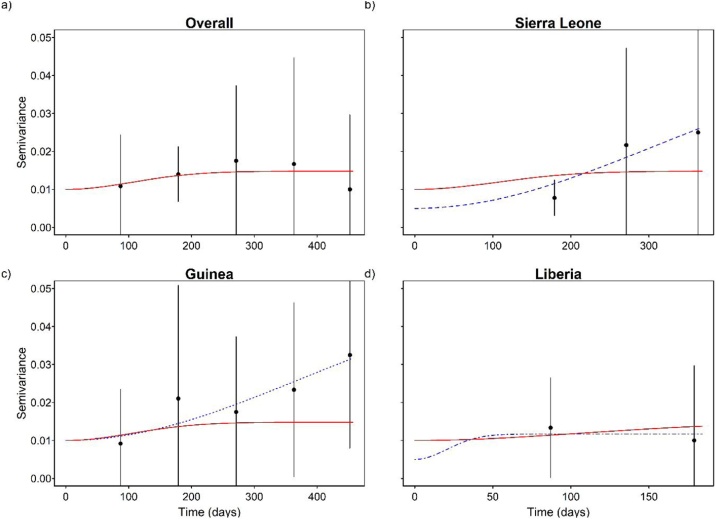
Semivariograms fitted with a Gaussian model to the residuals of quarter-specific CFR adjusted for imputations based on the Boosted Regression Tree model (BRT). The red line is the fitted model for the region as a whole and the blue lines are fitted models for individual countries (Sierra Leone, Guinea and Liberia). Note that the x-axes vary.

Quarter-specific results were reported for 2014 and 2015, this was because there were too few cases in late 2013 and early 2016 to be informative. The quarter-specific predicted and observed (observed CFR *qb*, as described in section 1.3, page 4, step 2 in the supplementary material) CFR also showed substantial heterogeneity and overall, both CFRs were lowest in the second quarter of 2014 (Figure 4a & b). The quarterly residuals of CFR showed no significant temporal clustering (D-W autocorrelation coefficient=-0.417, p = 0.454) (Figure 4c) as demonstrated by the flat temporal semivariograms of the residuals for both the whole region and each individual country (Figure 5).

## Discussion

Accounting for the entire EVD patient population including those with missing survival outcomes, this study represents the first detailed investigation of the spatiotemporal heterogeneity of CFR for the 2013–2016 Ebola epidemic in West Africa. This analysis characterises the magnitude of heterogeneity arising in a large epidemic and may be used to inform public health preparedness for future epidemics. In future, a similar analytical framework might be used to improve the situational awareness during ongoing outbreaks, such as that currently underway in the Democratic Republic of the Congo. This analytical framework could be even more informative in real-time if case referral routes were also recorded during outbreaks. Case referral routes would help disentangle whether survival outcomes are reported as promptly as deaths.

As observed previously, district-level CFR varied extensively across Sierra Leone, Guinea and Liberia (Garske et al., 2017, WHO Ebola Response Team, 2014). Indeed, Forna et al. identified district of origin as the most important predictor in explaining the observed variation in CFR (Forna et al., 2019). Here we found that even after accounting for district-level variation in the models, there remained significant (Moran’s I Index = 0.146, p = 0.03) regional and country-level (except for Sierra Leone) clustering of the CFR residuals and spatial independence was achieved only beyond 89.6 km (95% CI, 33.3–99.7 km). Thus, the distribution of CFR residuals was unlikely to have resulted from chance alone (regional Moran’s I Index p = 0.04). Other sources of spatial autocorrelation could be linked to the omission of potentially important predictors (Abdulhafedh, 2017, Miaou et al., 2003). However, because the residuals of the BRT model excluding district as a predictor showed even more significant autocorrelation (Moran’s I Index = 0.340, p < 0.001) and BRT is a non-parametric approach (i.e. captures both linear and non-linear relationships in data), we conclude that, at least for this dataset, the districts effects explain only some of the spatial variation in Ebola CFR.

In resource-constrained settings, as was the case in West Africa, the considerable spatial heterogeneity in district-level CFR could potentially be further explained by variations in case reporting practices across Sierra Leone, Guinea and Liberia. For instance, Liberia switched from an aggregate system of reporting to an individual-based system of reporting that could have led to preferential reporting of survival outcomes over time (McNamara, 2016). While in Sierra Leone, survival outcome reporting rates varied considerably throughout the epidemic (Garske et al., 2017). Inconsistent case reporting appears to be contributing to spatial heterogeneity in EVD CFR underscoring the need for the strengthening and harmonising of outbreak surveillance systems. Effective surveillance systems would improve data quality and subsequently, help us disentangle the contributions from the different mechanisms that may give rise to genuine differences in CFR estimates. During case reporting, a greater emphasis on survival outcome reporting would better serve future surveillance.

The kriging procedure yielded two advantages, as previously discussed by Carrat and Valleron (Carrat and Valleron, 1992). Firstly, CFR estimates were not constrained by the borders between administrative units, and sudden transitions in CFR between two neighbouring districts were avoided. Thus, these maps are robust to future boundary changes. Both the BRT model and kriging allowed us to interpolate CFR values for districts without observed survival data (e.g. Koubia, Lelouma, Labe and Mamou in Guinea) giving more complete CFR maps for the epidemic. In these districts, predicted CFR adjusted for imputation reflected the patterns observed in areas for which data were available.

Future work could compare these CFR estimates for every point on the map with additional localized epidemiological and demographic parameters. The identified spatial patterns can serve as a baseline for future preparedness and evaluation of control measures in the event of another Ebola outbreak in West Africa. The variability in the CFR across districts could inform the allocation of resources for control activities, with the possibility of focussing additional efforts in areas with higher CFRs (Kleinschmidt et al., 2001).

The findings from this study show no temporal pattern in CFR over the seven quarters of the epidemic, from January 2014 to September 2015, after accounting for the parameters in BRT model. This indicates that the BRT model captures all of the temporal correlation.

There are some limitations to this study which need to be considered when interpreting our results. Firstly, our findings do not explicitly reflect the variation in smaller administrative units such as chiefdoms, districts (the sub-administrative level in Liberia) and sub-prefectures in Sierra Leone, Liberia and Guinea, respectively. With more detailed data on patient residence, it would be interesting to compare our current results with those obtained based on the analysis of smaller administrative units. Secondly, unrecorded (including asymptomatic) EVD (Lefebvre et al., 2014) cases were not accounted for in our analysis. However, Forna et al. discussed the imperfect nature of the imputation process such that the uncertainty in CFR is reflected throughout our analysis (Forna et al., 2019). Using kriging based on estimated semivariograms for interpolation of CFR estimates for districts without survival data is not assumption-free. After including known predictors and adjusting the BRT model for imperfect sensitivity and specificity, in interpolating CFR, we assume that there are not unknown structural reasons for the districts without survival outcomes to be importantly different from districts with observed CFRs.

## Conclusion

Our results demonstrate that although BRT modelling accounted for most of the spatiotemporal variations and interactions in CFR, residual spatial autocorrelation remained. In contrast, residual temporal autocorrelation did not. Kriging was used to produce a more complete CFR map of the epidemic. This study adds to the literature that analysed the epidemiology of the 2013–2016 West African Ebola epidemic to inform future public health contingency planning, resource allocation and impact assessment. We also provide an analytical framework, which when used with other resources and key domain knowledge, could provide real-time support to the response to ongoing outbreaks, such as that currently underway in the Democratic Republic of the Congo.

## Ethical approval

Not required for this study.

## Financial support

This work was supported by the 10.13039/501100000867Commonwealth Scholarship Commission, UK Medical Research Council and 10.13039/501100000278Department for International Development (Centre funding) (grant number MR/R015600/1), National Institute of Health Research, and Imperial College Junior Research Fellowship, Sir Henry Dale Fellowship funded by the Royal Society and Wellcome Trust (grant number 213494/Z/18/Z).

## Conflict of interest statement

All authors: No reported conflicts of interest. All authors have submitted the ICMJE Form for Disclosure of Potential Conflicts of Interest. Conflicts that the editors consider relevant to the content of the manuscript have been disclosed.

## Contributors

Conceived and designed the experiments: AF ID PN* CAD*. Performed the experiments analysed the data: AF. Wrote the paper: AF ID PN* CAD*. * These authors contributed equally.
