# Supplementary material for: Spatiotemporal variability in case fatality ratios for the 2013–2016 Ebola epidemic in West Africa
Source: Int J Infect Dis. 2020 Apr;93:48–55. doi: 10.1016/j.ijid.2020.01.046 (PMC7191269; doi:10.1016/j.ijid.2020.01.046)
Supplement: Supplementary file 1 [file mmc1.docx]

**Supplementary material**

**Spatiotemporal Variability in Case Fatality Ratios for 2013–2016 Ebola epidemic in West Africa**

**Authors**: Alpha Forna^1^, MSc, Ilaria Dorigatti^1^, PhD, Pierre Nouvellet^1,2*^, PhD, and Christl A. Donnelly^1,3*^, ScD

**Affiliations: 1.** MRC Centre for Global Infectious Disease Analysis, Department of Infectious Disease Epidemiology, Imperial College London, London, United Kingdom.

**2.** School of Life Sciences, University of Sussex.

**3.** Department of Statistics, University of Oxford.

^*^These authors contributed equally.

**Correspondence to:** Alpha Forna

**Full Address**: MRC Centre for Global Infectious Disease Analysis, Department of Infectious Disease Epidemiology, Imperial College London, London, United Kingdom**.** 2 UG, St

Mary’s campus, Norfolk Place, London W2 1PG

**Email:** [a.forna16@imperial.ac.uk](mailto:a.forna16@imperial.ac.uk)

Table of Contents

[Table of Contents 2](#_Toc30175326)

[Details of Method 3](#_Toc30175327)

[1.1 Algorithm implemented for BRT hyperparameter tuning 3](#_Toc30175328)

[1.2 Algorithm implemented for spatial CFR prediction and residual estimation 3](#_Toc30175329)

[1.3 Algorithm implemented for temporal CFR prediction and residual estimation 4](#_Toc30175330)

[1.4 Algorithm implemented for BRT model imperfection 4](#_Toc30175331)

[Addition Results 5](#_Toc30175332)

[2.1 Geographical description of West Africa 5](#_Toc30175333)

[2.2 Moran’s I indices for CFR residuals 6](#_Toc30175334)

[2.3 Selecting a spatial model for the analysis 8](#_Toc30175335)

[2.4 Spatial semivariogram for observed CFR residuals 10](#_Toc30175336)

[2.5 Temporal semivariogram for observed CFR residuals 11](#_Toc30175337)

[2.6 Complete map of predicted CFR 12](#_Toc30175338)

[Sensitivity Analysis 13](#_Toc30175339)

[3.1 Directional semivariograms 13](#_Toc30175340)

[3.2 Comparing Gaussian and exponential models in mapping CFR 14](#_Toc30175341)

[References 16](#_Toc30175342)

# Details of Method

These algorithms follow closely those implemented in the preceding study (Forna et al., 2019). Detailed descriptions of Boosted Regression Trees (BRT) models and the required hyperparameter tuning are included in the preceding study and others (Dorigatti et al., 2018, Elith et al., 2008, Forna et al., 2019) .

## 1.1 Algorithm implemented for BRT hyperparameter tuning

For given values of $tc$, $lr$ and $bf$, and the proportions ($p$) of cases in training set, BRT models were built and validated using the following algorithm:

1. Generate the training set by randomly sampling without replacement the specified proportions of cases with death and survival outcomes.
2. Generate the out-of-sample validation set, consisting of the cases with observed survival outcomes excluded from the training set (step 1).
3. Build the BRT model through the gbm.step function in the ‘dismo’ package, using the training set built in step 1, the specified $tc$, $lr$, $bf$, 10-fold cross-validation and increasing the model in steps of 50 trees at each iteration, using survival outcome (i.e. death or survival) as the response variable and gender, health care worker, health care worker position, bleeding gums, bleeding nose, bleeding skin, bleeding stool, bleeding urine, haematemesis, vomiting, cough, bleeding from other sites, bleeding injection site, bleeding vagina, unexplained bleeding, fever, jaundice, painful eyes, rash, unconsciousness, fatigue, anorexia, vomiting, diarrhoea, headache, abdominal pain, muscle pain, joint pain, chest pain, coughing blood, difficult breathing, conjunctivitis, sore throat, confused, hiccup, difficulty swallowing, case classification, quarter, age, delay, current hospitalisation, districts and country of origin as the predictors. The optimal number of trees minimising the holdout (cross-validation) deviance is computed.
4. Use the BRT model built in step 3 to predict the survival outcomes of the cases in the validation set. For each subject $i$ in the validation set, the BRT model gives the probability $p$ that subject $i$is dead.
5. Define the cut-off threshold $p_{T}$ , by which cases with $p_{i}$ >$p_{T}$ are classified as dead or alive otherwise. We chose cut-off values giving equal sensitivity and specificity using the optimal.threshold function in the PresenceAbsence package.
6. Use the cut-off threshold $p_{T}$ computed in step 5 to classify cases in the validation set as dead or alive.
7. Compute the sensitivity (proportion of deaths correctly classified), specificity (proportion of survivals correctly classified) and percentage of predictions correctly classified (PCC) in the validation set and the area under the receiver operating characteristic curve (AUC).

The choice of optimising the cut-off thresholds to have equal sensitivity and specificity in step 5 was made to avoid introducing bias in CFR estimated predictions.

## 1.2 Algorithm implemented for spatial CFR prediction and residual estimation

The median CFR predictions were calculated from 1,000 realisations of steps 1 – 7 (as list above), followed by steps 8 to 14 below:

1. Use the BRT model built in step 4 to predict for all cases in the dataset.
2. Use the cut-off threshold $p_{T}$ computed in step 6 to classify imputed cases as dead or alive.
3. Calculate the predicted CFR for individual cases.
4. Adjust the predictions for model imperfection (see section 1.4)
5. Calculate the median prediction for all individuals within a particular district (*i)***.**
6. Calculate the residual for each district. Residual (*i)* = Observed CFR (*i*) – Median predicted CFR for all individuals in district (*i).* Observed CFR (*ia*) was estimates without adjusted imputation for cases with unknown survival outcomes and Observed CFR (*ib*) was estimated with adjusted imputation for cases with unknown survival outcomes. Thus, Residual(*ia*) and Residual (*ib*) were estimated.
7. Calculate the semivariogram for these spatial residuals (i.e. *ia* and *ib*).

Confidence interval

Confidence intervals around the median CFR predictions and residuals were calculated by bootstrapping as previously described (Forna et al., 2019). Using the optimal tuning parameter set ($tc$ = 27, $lr$ = 0.001, $bf$ = 0.75), the 95% CI of the CFR estimates with imputation were calculated as the 2.5-97.5 percentiles of 1,000 random (i.e. sampling the data each time with replacement) realisations of the algorithm.

## 1.3 Algorithm implemented for temporal CFR prediction and residual estimation

The median temporal predictions and residuals were calculated following steps 1–11 (appendix p 3-4) followed by steps 1 to 3 below:

1. Calculate the median prediction for all individuals by quarter.
2. Calculate the residual for each quarter. Residual (*q)* = Observed CFR (*q*) – Median predicted CFR per quarter (*q).* Observed CFR (*qa*) was estimates without adjusted imputation for cases with unknown survival outcomes and Observed CFR (*qb*) was estimated with adjusted imputation for cases with unknown survival outcomes. Thus, Residual(*qa*) and Residual (*qb*) were estimated.
3. Calculate the semivariogram for these temporal residuals (i.e. *qa* and *qb).*

Confidence interval

Confidence intervals around the median CFR predictions and residuals were calculated by bootstrapping as previously described (Forna et al., 2019). Using the optimal tuning parameter set (tc = 27, lr = 0.001, bf = 0.75), the 95% CI of the CFR estimates with imputation were calculated as the 2.5-97.5 percentiles of 1,000 random realisations of the algorithm.

## 1.4 Algorithm implemented for BRT model imperfection

The median CFR predictions at step 11 were adjusted for imperfect BRT model imputation using following steps 1,2,3 and 4 below:

1. Generate cases with just the imputed death outcomes.
2. Use the dead cases in step 1, the total number of cases in the imputed data and tp function in the RSurveillance package to estimate the inferred CFR.
3. Multiply the number of cases in the imputed data by the inferred CFR to get the number of inferred deaths $f_{T}$.
4. Adjusted predicted CFR is the ratio of the sum of $f_{T}$ and the number of deaths in data with known survival outcomes, divided by the sum of all deaths and survivals in the complete data (i.e. data with both imputed and observed outcomes).

Confidence interval

Although the tp function of the “RSurveillance” package provides parametric approaches to estimate confidence intervals (Sergeant, 2016), in our implementation, confidence intervals around the median adjusted CFR estimates were calculated by non-parametric bootstrapping (Forna et al., 2019). Using the optimal tuning parameter set ($tc$ = 27, $lr$ = 0.001, $bf$= 0.75), the 95% CI of the adjusted CFR estimates were calculated as the 2.5-97.5 percentiles of 1,000 random realisations of the algorithm.

# Addition Results

## 2.1 Geographical description of West Africa

The map shows the 63 districts of Sierra Leone, Guinea and Liberia included in the analysis. The outbreak started in Gueckedou a district (prefecture) in Guinea which became the initial epicentre of the epidemic (Timothy et al., 2019). As seen in Figure 1, Gueckedou borders districts in both Sierra Leone (i.e. Kono and Kailahun) and Liberia (i.e. Lofa) and beyond the political boundaries, inhabitants (the Kissi people) in these districts share a common language and similar cultures (Wilkinson and Fairhead, 2017). This inter-country homogeneity together with other factors contributed greatly to magnitude of the West African Ebola epidemic.


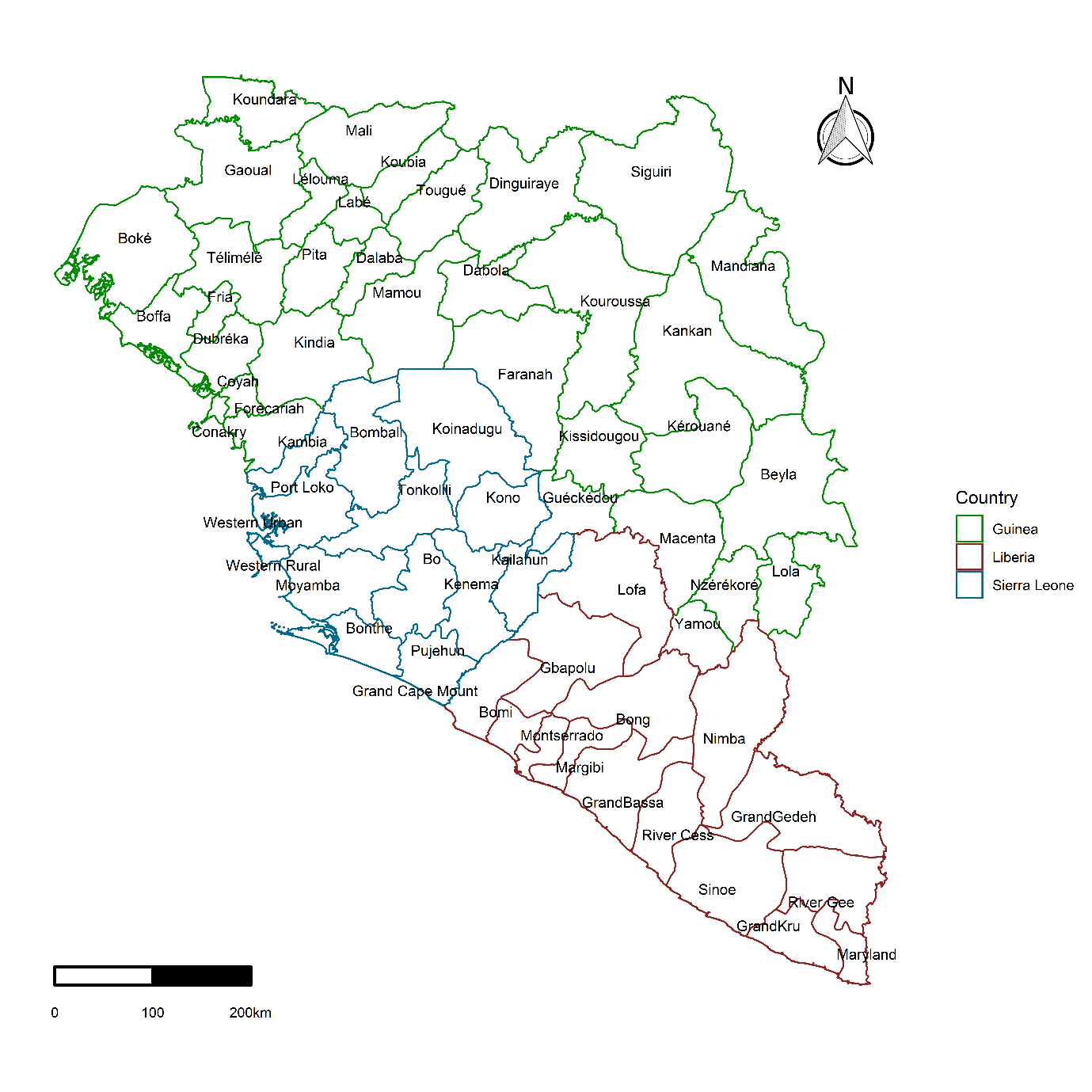


**Figure 1.** The geographical location and distribution of administrative districts in Sierra Leone, Guinea and Liberia.

## 2.2 Moran’s I indices for CFR residuals

Table 1 shows the local autocorrelation for districts in Sierra Leone, Guinea and Liberia. We consider districts with p-values <0.05 (i.e. p-values shaded red) as districts for which CFR is spatially correlated to that of neighbouring districts. Here we fitted the BRT model with and without the district predictor in the model.

**Table 1.** Spatial autocorrelation analysis for case fatality ratio (CFR) residuals in Sierra Leone, Guinea and Liberia from 2013 to 2016, with/without district predictor in the BRT model. The global Moran’s I is for the three countries combined. The two-sided p-values in red are statistically significant, <0.05.

| District | With district as a predictor in the BRT model | | Without district as a predictor in the BRT model | |
| --- | --- | --- | --- | --- |
|  | Moran's I | p-value | Moran's I | p-value |
| Global | 0.146 | 0.033 | 0.348 | <0.001 |
| Guinea |  |  |  |  |
| BOFFA | -0.019 | 0.969 | 0.011 | 0.933 |
| BOKE | -0.016 | 0.978 | 0.011 | 0.933 |
| FRIA | -0.019 | 0.969 | -0.055 | 0.850 |
| CONAKRY | -0.084 | 0.784 | 0.475 | 0.067 |
| DABOLA | 0.158 | 0.588 | 0.115 | 0.682 |
| DINGUIRAYE | -0.133 | 0.647 | -0.147 | 0.610 |
| FARANAH | 0.013 | 0.925 | -0.036 | 0.925 |
| KISSIDOUGOU | 0.088 | 0.735 | 0.420 | 0.157 |
| KEROUANE | 0.536 | 0.056 | 0.812 | 0.004 |
| KANKAN | 0.207 | 0.441 | 0.032 | 0.874 |
| KOUROUSSA | 0.273 | 0.283 | 0.222 | 0.380 |
| SIGUIRI | 0.095 | 0.663 | 0.177 | 0.438 |
| COYAH | 0.536 | 0.056 | 0.520 | 0.064 |
| DUBREKA | 0.007 | 0.941 | 0.217 | 0.423 |
| FORECARIAH | -0.101 | 0.762 | -0.084 | 0.808 |
| KINDIA | 0.187 | 0.484 | -0.004 | 0.974 |
| TELIMELE | -0.181 | 0.521 | -0.147 | 0.610 |
| MALI | -0.118 | 0.741 | 0.004 | 0.949 |
| TOUGUE | 0.062 | 0.781 | 0.078 | 0.737 |
| DALABA | -0.084 | 0.784 | 0.078 | 0.737 |
| PITA | 0.207 | 0.441 | -0.034 | 0.945 |
| BEYLA | -0.908 | <0.001 | 0.361 | 0.122 |
| GUECKEDOU | -0.165 | 0.516 | 0.411 | 0.079 |
| LOLA | 0.013 | 0.926 | 0.627 | 0.026 |
| MACENTA | 0.173 | 0.486 | 0.904 | 0.001 |
| NZEREKORE | 0.885 | 0.002 | 0.910 | 0.001 |
| YAMOU | 0.173 | 0.486 | 0.222 | 0.380 |
| Sierra Leone | |  |  |  |
| KAILAHUN | -0.685 | 0.019 | 0.003 | 0.952 |
| KENEMA | 0.772 | 0.003 | 0.437 | 0.091 |
| KONO | 0.403 | 0.118 | 0.344 | 0.180 |
| BOMBALI | 0.094 | 0.708 | 0.479 | 0.087 |
| KAMBIA | 0.013 | 0.925 | 0.698 | 0.008 |
| KOINADUGU | 0.441 | 0.137 | 0.785 | 0.009 |
| PORTLOKO | 0.045 | 0.837 | 0.709 | 0.012 |
| TONKOLILI | 0.289 | 0.292 | 0.930 | <0.001 |
| BO | 0.174 | 0.535 | 0.704 | 0.019 |
| BONTHE | 0.013 | 0.925 | 0.212 | 0.399 |
| MOYAMBA | -0.457 | 0.061 | -0.015 | 0.981 |
| PUJEHUN | 0.257 | 0.311 | 0.212 | 0.399 |
| WESTERN | 0.013 | 0.925 | -0.102 | 0.733 |
| Liberia | - |  |  |  |
| BOMI | 0.729 | 0.010 | -0.012 | 0.995 |
| BONG | 0.077 | 0.762 | 0.057 | 0.812 |
| GBAPOLU | 0.012 | 0.926 | 0.072 | 0.781 |
| GRANDCAPEMOUNT | 0.852 | 0.003 | 0.210 | 0.437 |
| GRANDBASSA | -0.362 | 0.140 | 0.215 | 0.351 |
| GRANDGEDEH | -0.052 | 0.894 | -0.388 | 0.194 |
| GRANDKRU | 0.143 | 0.586 | 0.299 | 0.276 |
| LOFA | 0.408 | 0.142 | 0.812 | 0.004 |
| MARGIBI | 0.199 | 0.384 | 0.170 | 0.455 |
| MARYLAND | -0.019 | 0.969 | -0.144 | 0.573 |
| MONTSERRADO | 0.223 | 0.436 | 0.252 | 0.383 |
| NIMBA | 0.105 | 0.680 | -0.329 | 0.274 |
| RIVERCESS | 0.028 | 0.885 | 0.162 | 0.562 |
| RIVERGEE | 0.079 | 0.713 | -0.020 | 0.964 |
| SINOE | -0.067 | 0.808 | 0.035 | 0.853 |

## 2.3 Selecting a spatial model for the analysis

To identify the best theoretical spatial model to fit to the empirical semivariogram, we estimated the Akaike’s Information Criterion (AIC) for 5 model types (i.e. Linear, Cauchy, Exponential, Matern and Gaussian). Table 2 shows the AIC values for each spatial model and because the Gaussian model yielded the smallest AIC, we used the Gaussian model for subsequent analysis.

Figure 2 shows different parametric forms for semivariograms (i.e. linear, Cauchy, exponential and Matern) fitted to empirical semivariogram of Sierra Leone, Guinea and Liberia combined. The Cauchy, exponential and Matern models fit well to empirical semivariogram. The range [89.6 km (95% CI, 33.3–99.7 km)] of the Gaussian model is more precisely estimated than that of the exponential model [112.0 km (95% CI, 0–112.0 km)]. These results together with the smaller AIC for the Gaussian model, affirms our choice of using the Gaussian model for subsequent analysis.


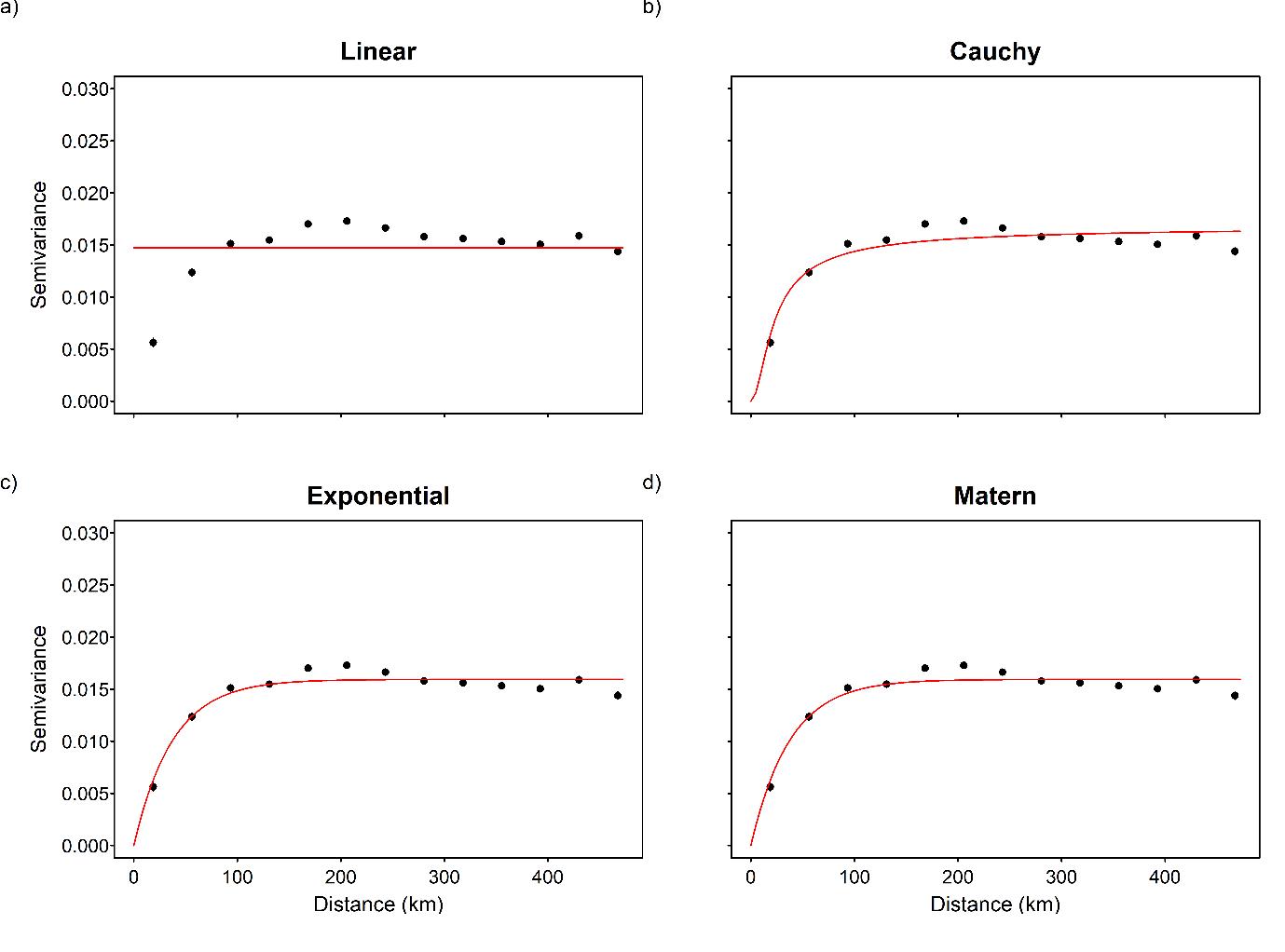


**Figure 2.** Different parametric forms for semivariograms fitted to the residual empirical semivariogram for Sierra Leone, Guinea and Liberia combined.

**Table 2.** Spatial semivariogram models fitted to overall district level CFR residuals.

| Spatial models | AIC |
| --- | --- |
| Linear | -46.8112 |
| Cauchy | -62.0444 |
| Exponential | -62.9812 |
| Matern | -62.9812 |
| Gaussian | -64.2103 |

## 2.4 Spatial semivariogram for observed CFR residuals

Figure 3 shows the spatial semivariogram for observed CFR residuals (*iq*) without the adjusted imputation for cases with unknown outcomes. These residuals also show spatial autocorrelation. However, because we excluded cases with unknown outcomes, the observed residuals are not fully representative of the patient mix. Thus, BRT imputation makes the CFR estimates more representative and allows for better characterisation of the spatiotemporal heterogeneity in district-level CFR. We did not proceed to generate kriged maps for these residuals.


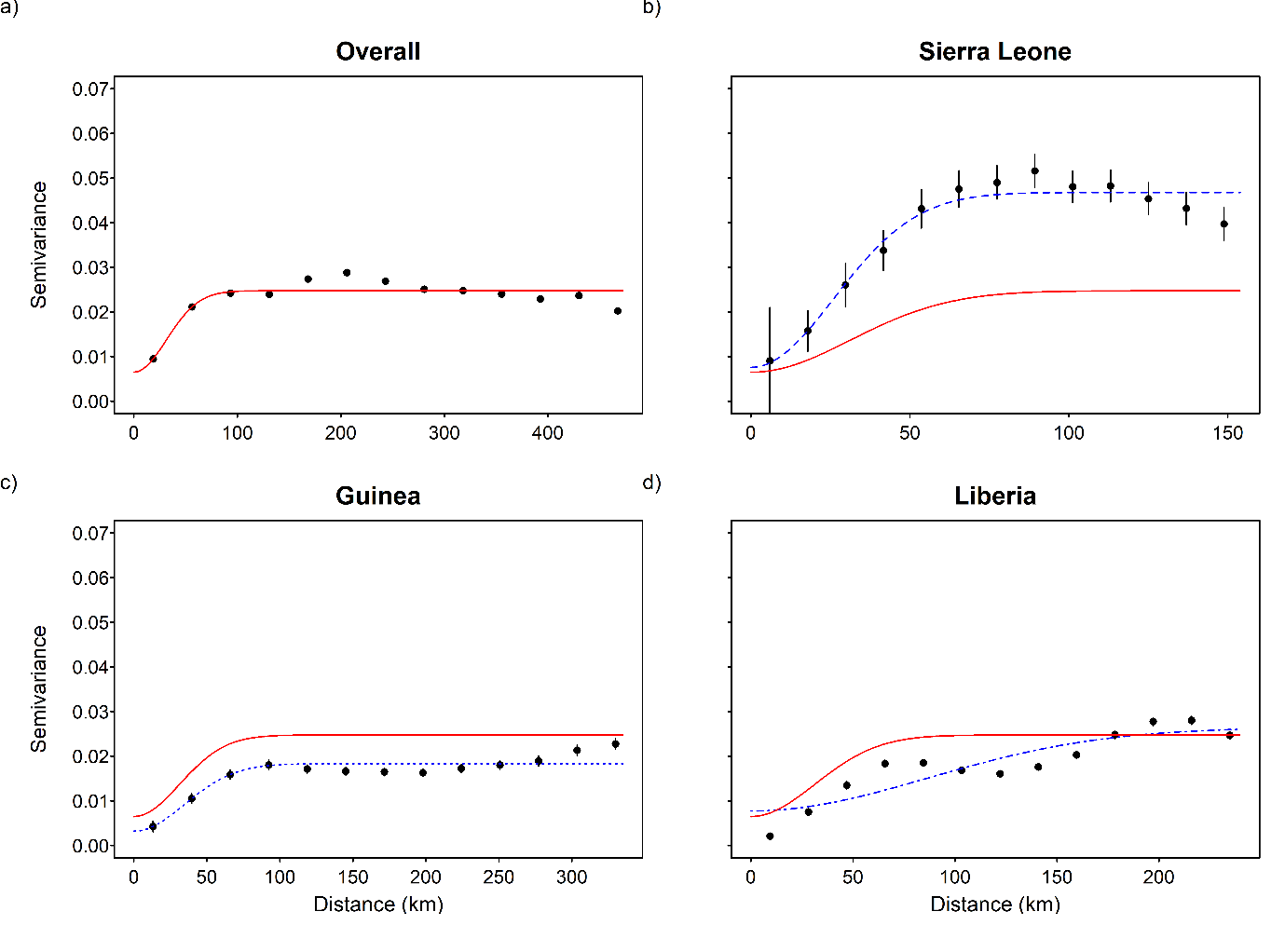


**Figure 3.** Semivariograms fitted to the residuals of Observed CFR. Red line fitted model for overall, blue lines fitted models for individual countries. Note that the x-axes vary.

## 2.5 Temporal semivariogram for observed CFR residuals

Figure 4 shows the temporal semivariogram for observed CFR residuals (*qq*) without the adjusted imputation for cases with unknown outcomes. Similar to the residuals for observed CFR with adjusted imputations, these residuals do not show temporal autocorrelation.


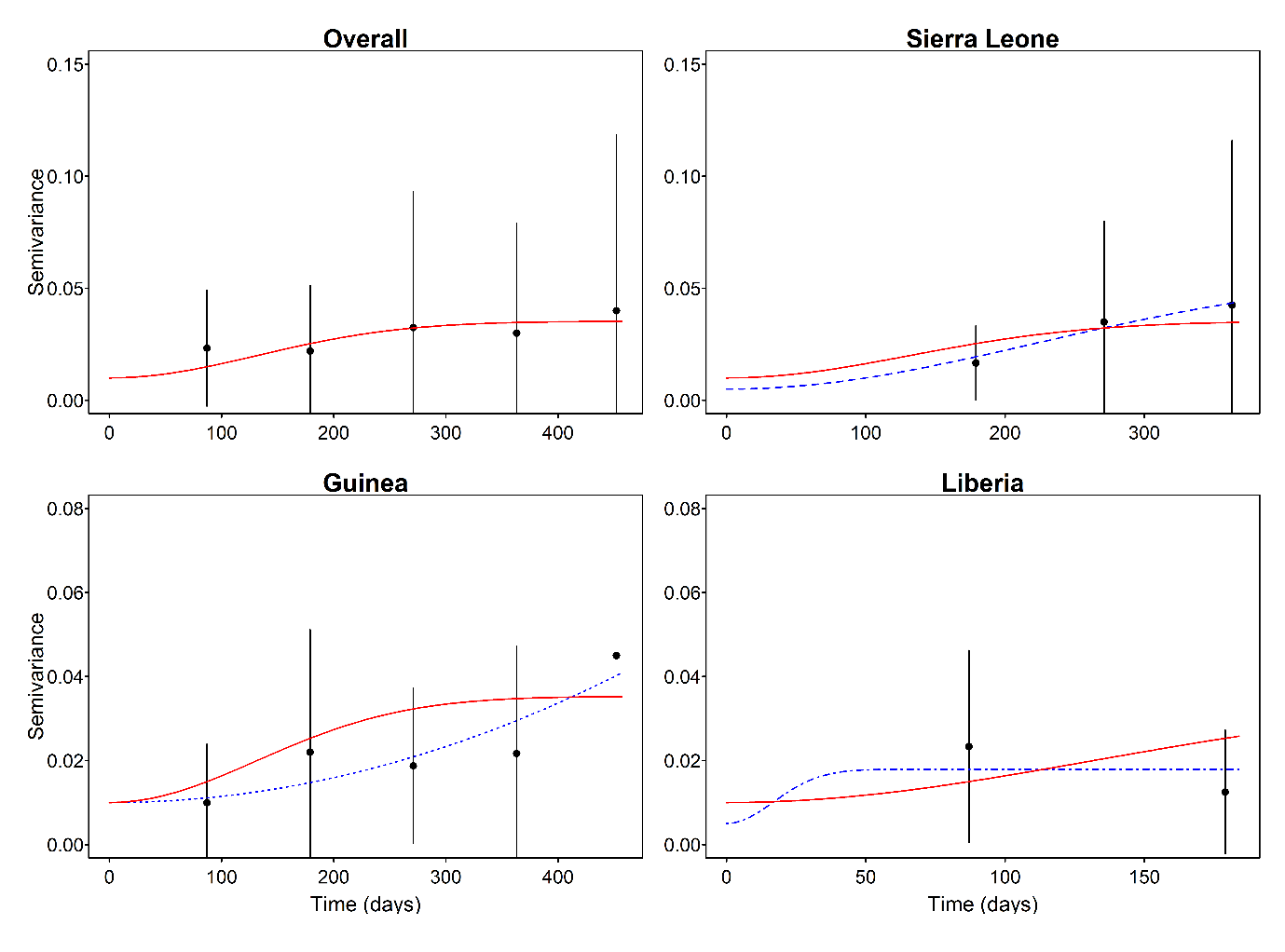


**Figure 4.** Semivariograms fitted to the residual of observed CFRs. Red line fitted model for overall, blue lines fitted models for individual countries. Note that the x-axes vary.

## 2.6 Complete map of predicted CFR

Figure 5a show the isopleth (i.e. continuous grid) of the predicted CFR adjusted for imputation. The kriged residuals were added to these predictions to produce the complete unbiased map presented in Fig 3c in the main manuscript. Regions with relatively high uncertainty (standard deviation) show districts for which data were unavailable (Figure 5b).


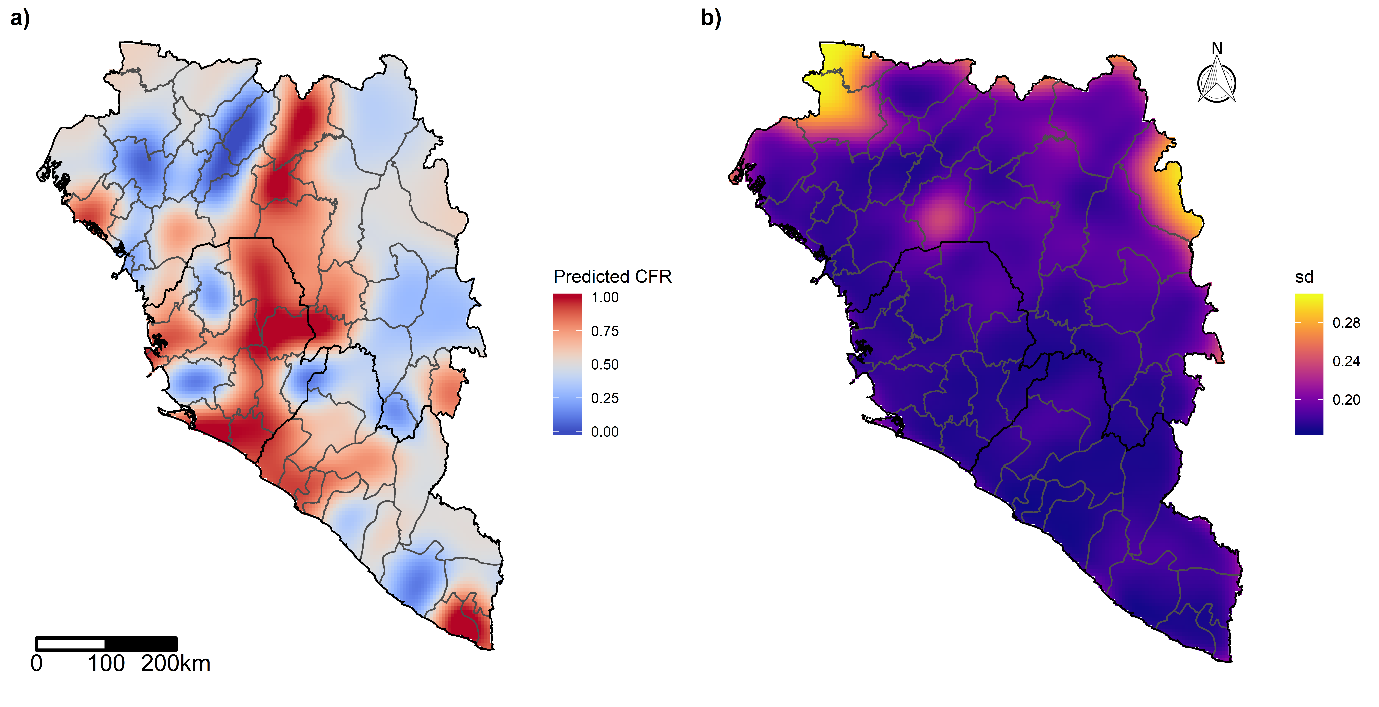


**Figure 5.** Isopleth map for predicted CFR adjusted for imputation.

# Sensitivity Analysis

## 3.1 Directional semivariograms

Coupled with distance, direction could also have influenced the semivariance between districts (Brooker et al., 2004). To investigate anisotropy, we estimated semivariograms for different directions, 0^0^, 45^0^, 90^0^ and 135^0^ (Figure 6). The directional semivariograms did not appear to exhibit marked differences; thus, we assumed an isotropic spatial process and report omnidirectional semivariograms throughout this study.

To gain more insight into the isotropy properties, we perform a nonparametric hypothesis test of isotropy using the georeferenced district-level CFR residuals. We used the “GuanTestGrid” function from the R package “spTest” to carry out a nonparametric hypothesis test (Weller, 2018). The resulting insignificant p-value (0.33) confirmed conclusions drawn from the directional semivariograms that the dependence between district-level CFR residuals is a function of only the distance and not the direction of the spatial separation between sampling locations.


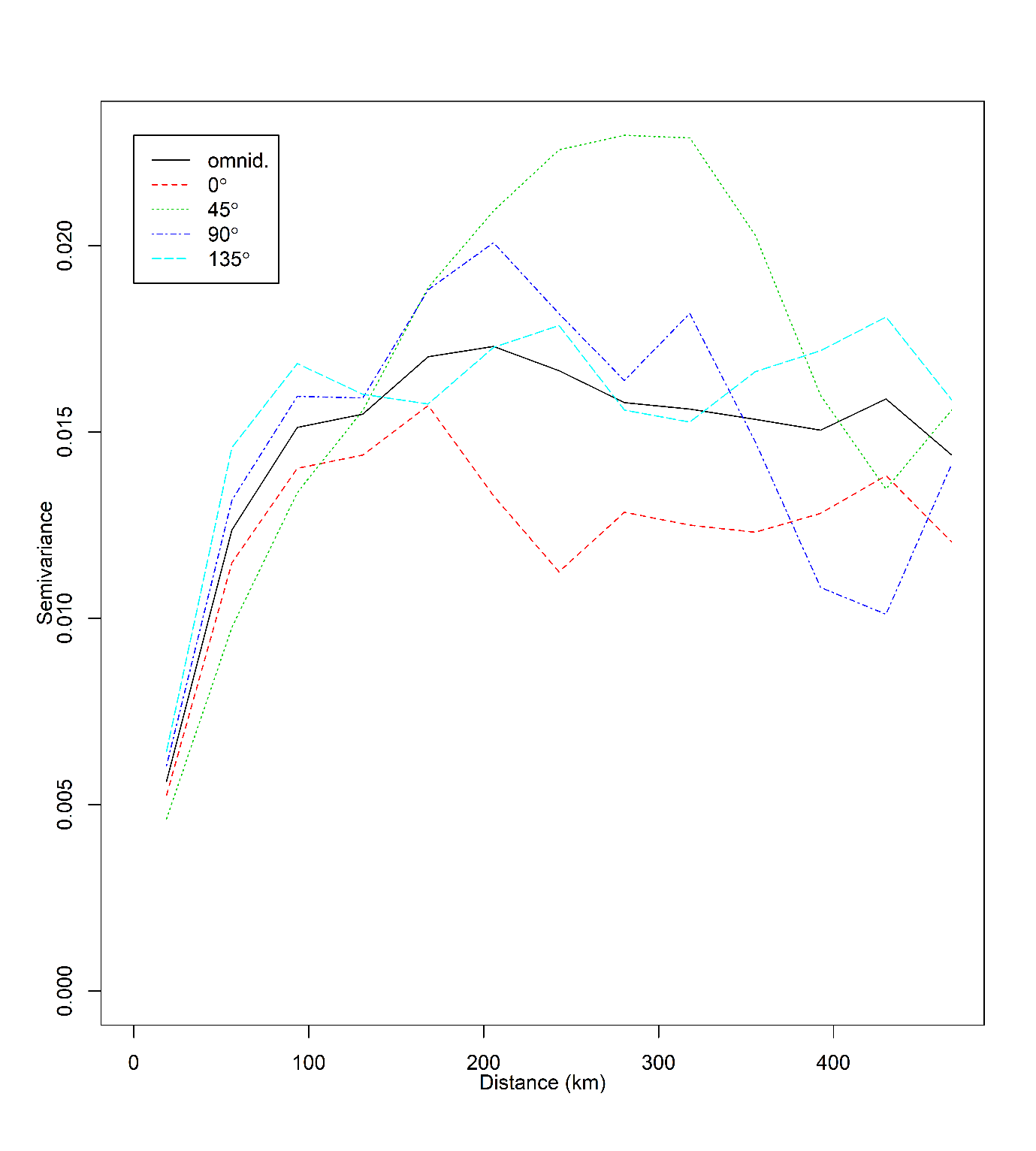


**Figure 6.** Directional semivariograms for overall (i.e. Sierra Leone, Guinea, and Liberia).

## 3.2 Comparing Gaussian and exponential models in mapping CFR

Although we fitted a Gaussian model to the semivariograms in the main analysis, as a sensitivity analysis, we show that maps of residuals and kriged CFRs are similar for both the Gaussian and exponential models (Figure 7 and Figure 8).


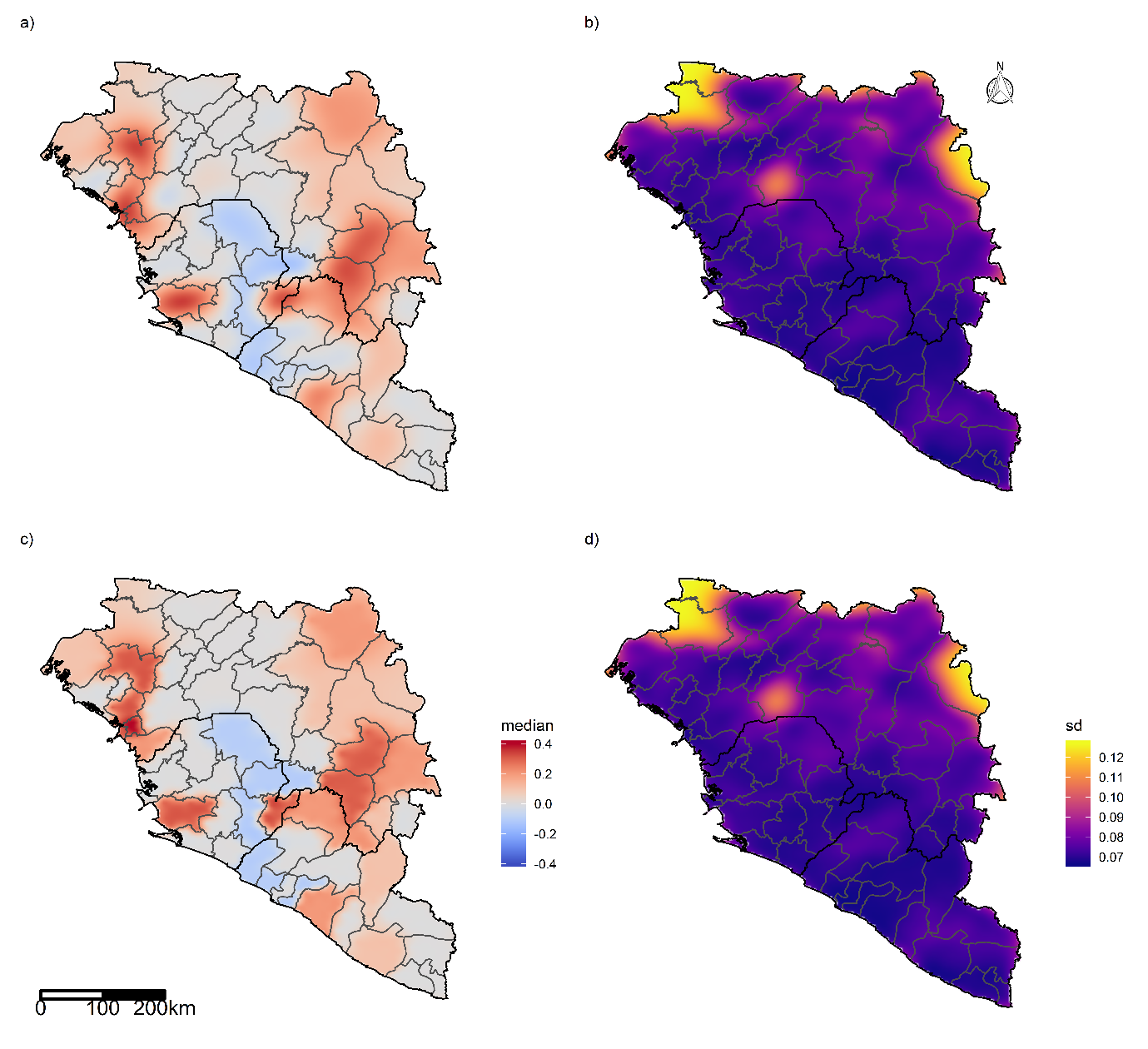


**Figure 7**: Isopleth maps for kriged residuals. [a–b] median and standard deviation for the overall semivariogram fitted with a Gaussian model. [c–d] median and standard deviation for the overall semivariogram fitted with an exponential model.


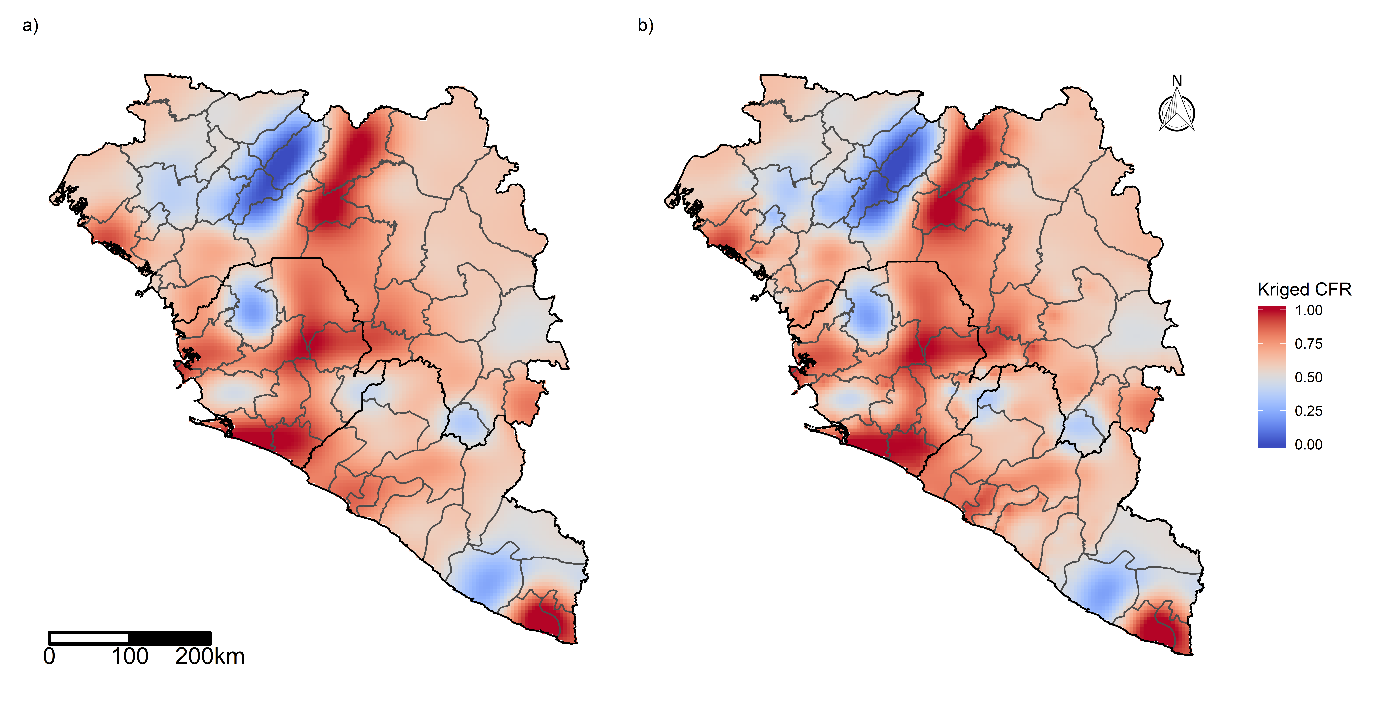


**Figure 8:** Isopleth map for kriged CFRs (i.e. predicted CFR adjusted for imputation plus the kriged residuals). a) Kriged CFR for semivariogram fitted with a Gaussian model. b) Kriged CFR for semivariogram fitted with an exponential model.

# References

Brooker S, Kabatereine N, Tukahebwa E, Kazibwe F. Spatial analysis of the distribution of intestinal nematode infections in Uganda. Epidemiology & Infection 2004;132(6):1065-71.

Dorigatti I, Donnelly C, Laydon D, Small R, Jackson N, Coudeville L, et al. Refined efficacy estimates of the Sanofi Pasteur dengue vaccine CYD-TDV using machine learning. Nature communications 2018;9(1):3644.

Elith J, Leathwick JR, Hastie T. A working guide to boosted regression trees. J Anim Ecol 2008;77(4):802-13.

Forna A, Nouvellet P, Dorigatti I, Donnelly CA. Case fatality ratio estimates for the 2013 – 2016 West African Ebola epidemic: application of Boosted Regression Trees for imputation. Clin Infect Dis 2019.

Sergeant E. Package ‘ RSurveillance ’. 2016.

Timothy JW, Hall Y, Akoi-Boré J, Diallo B, Tipton TR, Bower H, et al. Early transmission and case fatality of Ebola virus at the index site of the 2013–16 west African Ebola outbreak: a cross-sectional seroprevalence survey. The Lancet Infectious Diseases 2019;19(4):429-38.

Weller ZD. spTest: An R Package Implementing Nonparametric Tests of Isotropy. Journal of Statistical Software 2018;83(1):1-24.

Wilkinson A, Fairhead J. Comparison of social resistance to Ebola response in Sierra Leone and Guinea suggests explanations lie in political configurations not culture. Critical Public Health 2017;27(1):14-27.
